# A Snapshot of The Tumor Microenvironment in Colorectal Cancer: The Liquid Biopsy

**DOI:** 10.3390/ijms20236016

**Published:** 2019-11-29

**Authors:** Mercedes Herrera, Cristina Galindo-Pumariño, Vanesa García-Barberán, Cristina Peña

**Affiliations:** 1 Department of Oncology-Pathology, Karolinska Institutet, 17177 Stockholm, Sweden; mercedes.herrera@ki.se; 2 Medical Oncology Department, Instituto Ramón y Cajal de Investigación Sanitaria (IRYCIS), Alcalá University, 28034 Madrid, Spain; crisgpuma@gmail.com; 3 Centro de Investigación Biomédica en Red de Cancer (CIBERONC), 28029 Madrid, Spain; 4 Laboratorio de Oncología Molecular, Hospital Clínico San Carlos, Instituto de Investigación Sanitaria San Carlos (IdISSC), 28040 Madrid, Spain

**Keywords:** colorectal cancer, tumor microenvironment, liquid biopsy, exosomes

## Abstract

The molecular profile of liquid biopsies is emerging as an alternative to tissue biopsies in the clinical management of malignant diseases. In colorectal cancer, significant liquid biopsy-based biomarkers have demonstrated an ability to discriminate between asymptomatic cancer patients and healthy controls. Furthermore, this non-invasive approach appears to provide relevant information regarding the stratification of tumors with different prognoses and the monitoring of treatment responses. This review focuses on the tumor microenvironment components which are detected in blood samples of colorectal cancer patients and might represent potential biomarkers. Exosomes released by tumor and stromal cells play a major role in the modulation of cancer progression in the primary tumor microenvironment and in the formation of an inflammatory pre-metastatic niche. Stromal cells-derived exosomes are involved in driving mechanisms that promote tumor growth, migration, metastasis, and drug resistance, therefore representing substantial signaling mediators in the tumor-stroma interaction. Besides, recent findings of specifically packaged exosome cargo in Cancer-Associated Fibroblasts of colorectal cancer patients identify novel exosomal biomarkers with potential clinical applicability. Furthermore, additional different signals emitted from the tumor microenvironment and also detectable in the blood, such as soluble factors and non-tumoral circulating cells, arise as novel promising biomarkers for cancer diagnosis, prognosis, and treatment response prediction. The therapeutic potential of these factors is still limited, and studies are in their infancy. However, innovative strategies aiming at the inhibition of tumor progression by systemic exosome depletion, exosome-mediated circulating tumor cell capturing, and exosome-drug delivery systems are currently being studied and may provide considerable advantages in the near future.

## 1. Introduction

A tissue biopsy is normally used for diagnosis and monitoring procedures. However, this method has limitations in cancers due to intratumor heterogeneity. As a single biopsy cannot show the complexity of tumor composition, multiple biopsies would be needed, which is uncomfortable for the patient and, anyway, is not ethically approved. In addition, in some cases and depending on location or accessibility, there is a risk of spreading malignant cells or impeding surgery [1].

In this context, liquid biopsy is posited as a new method for early detection and tracking of biomarkers, essentially in blood. Liquid biopsy is considered a minimally invasive method for obtaining biological fluids including urine, pleural effusion, ascites, cerebrospinal fluid and, predominantly, peripheral blood [2,3]. Liquid biopsy is used to obtain compounds such as circulating tumor cells (CTCs), circulating free DNA, circulating free DNA that derives from the tumor (ctDNA), circulating free RNAs (including non-coding RNAs), exosomes and other circulating extracellular vesicles [2,3,4]. This review focuses on the tumor microenvironment components which are detected in the blood samples of colorectal cancer (CRC) patients. 

Free circulating DNA can be found in both cancer patients and healthy individuals but is higher in cancer patients [5,6]. This can be explained by, under normal conditions, the release of DNA by cells suffering apoptosis or necrosis. However, clearance processes also reduce cell debris and DNA [1,7]. In cancer this cell turnover is increased: as the tumor grows, ctDNA release also increases [7,8], which is associated with bad prognoses such as decreased overall survival [9,10]. 

As mutations found in ctDNA are not always the same as those first found in primary tumors, ctDNA can give an idea of the sub-populations of tumor cells present in tumors and how they might turn out to be resistant to treatment [1,5]. For instance, “KRAS proto-oncogene, GTPase” KRAS mutations can be detected in ctDNA even though the biopsy was KRAS wild type at diagnosis: these can lead to resistance to therapy and tumor relapse [2,7]. 

CTCs can be found in the blood in early tumor formation [5]. Levels of CTCs in peripheral blood are associated with decreased progression-free survival and overall survival [10,11]. Isolation is possible after enrichment steps, basically using antibody-conjugated particles, while detection is based on highly sensitive technologies such as microchips, PCR assays, or microscopic systems [2,4,5]. CTC detection has been used for response prediction in lung, colon, breast, and pancreas cancer, as reported in several studies [11,12,13]. Moreover, CTCs can also be a source of ctDNA detected by liquid biopsy, but this is not considered the primary source due to the low level of CTCs present in blood [1]. 

Interestingly, while searching for new cancer biomarkers based on blood samples, recent studies describe new types of circulating non-tumoral cells in cancer patients, which are not detected in healthy controls. These cells correspond to macrophage-like cells, tumor endothelial or progenitor endothelial cells and cancer-associated fibroblasts. Their diagnostic, prognostic, and treatment response values have also been determined [14]. 

Exosomes are vesicles of 40–150 nm in diameter that hold proteins, lipids, small DNAs and RNAs [3]. All cell types produce exosomes, whose content varies widely between cell types and individual status [15,16]. These vesicles can recognize target cells, be internalized and release their content to induce a response [16]. As the tumor grows, the content of the exosomes released by cancer cells will also vary, reflecting both the spatial and the temporal heterogeneity of the malignancy [16].

Although DNA can be found in exosomes, most studies target RNAs. Exosomes contain a small proportion of mRNAs, but mainly non-coding RNAs [17]. MicroRNAs may be the most frequently studied small RNA in exosomes, but are not the most abundant type [15]. For example, long non-coding RNAs (lncRNAs) are emerging as a relevant new molecule type found in exosomes [18]. Exosome content has been analyzed when looking for potential biomarkers for diagnosis and prognosis. Several studies of various cancer types have proposed micro RNAs (miRNAs) as candidates for early detection or prognosis [19,20]. 

Tissue biopsies, though gold standard, still involves difficulties for clinicians and risk to the patient, which is a major reason to keep studying liquid biopsy. Detection of newly acquired mutations or therapeutic biomarkers during disease monitoring will help clinicians to adapt treatment to the evolution of the tumor, which helps the patient and personalizes treatment. Detection of ctDNA, CTCs or exosomes is considered a real-time perception of disease [3,9,21,22]. For example, in colorectal cancer, new KRAS mutation, causing anti-Epithelial Growth Factor Receptor (EGFR) therapy to fail, can be detected in circulating free DNA several months before detection of progression [7].

Technical complexity in detection is still an issue. Even though this is a sensitive technology, it is mainly used for small-scale research and is not established in routine analyses [7]. As contamination during CTC isolation is practically unavoidable, other cell types are also captured [3].

It has been observed that tumor cells communicate not only with other malignant cells but also with the components of the tumor microenvironment. Extracellular matrix, blood vessels, immune cells, and fibroblasts are the main components of what is known as the tumor microenvironment [23,24]. 

Under normal conditions, activated fibroblasts are characterized by the expression of activation markers, like α-Smooth Muscle Actin (α-SMA) or Platelet derived Growth Factorα (PDGFRα), and participate in the wound-healing process [25]. While in normal tissue activation of fibroblasts is reverted, in tumors fibroblasts appear to be permanently activated and are known as Cancer-Associated Fibroblasts (CAFs), which form a heterogeneous group of cells with different activation markers [25]. CAFs actively participate in Extracellular Matrix (ECM) deposition and remodeling, which are related to disease progression [24,26,27]. CAFs and tumor cells interact, due, in part, to secreted exosomes [23].

In addition, in the tumor microenvironment context, endothelial cells play the role of communicating tumor cells with surrounding areas by generating new vascular networks or modifying pre-existing vessels, thus conditioning tumor oxygen and nutrient supply [28]. Since endothelial cells, as well as CAFs and tumor cells, affect immune cell recruitment within the tumor, it can be assumed that the tumor microenvironment will condition immune response. T-cell activation can end up by either stimulating or inhibiting the immune system, depending on many factors, such as tumor antigen production, regulation of inhibitory ligands, angiogenesis, CAFs’ chemokine secretion, etc. [28]. In this intercommunication, exosome release and uptake also play a crucial role that will be reviewed in detail. 

## 2. Cross-Talk between Tumor and Microenvironmental Cells by Exosome Transference 

Cell-to-cell communication is carried out through different mechanisms: direct cell-cell and cell-matrix interactions, via soluble ligands, extracellular particles (non-membranous, such as exomeres) and extracellular vesicles (EV).

Extracellular vesicles are surrounded by a lipid bilayer and transfer bioactive molecules (proteins, lipids, DNA, mRNA, ncRNA, and metabolites) to recipient cells, modifying functions in these cells. These vesicles have been isolated from numerous body fluids, such as plasma, saliva, urine, cerebrospinal fluid, etc. [29]. Although many different names are used in the literature, referring to size, cell or tissue of origin, functions, etc., there are three main subgroups of EV based on biogenesis, size and membrane composition: (a) apoptotic bodies, (b) microvesicles released by budding from the plasma membrane (generally referring to 150–1000 nm), and (c) small exosomes of endosomal origin (30–150 nm).

In recent years, exosomes have been extensively studied. They are secreted by different cell types, such as tumoral cells, immune cells, and stromal cells, and modify functions in diverse recipient cells. In the oncological process, they play important roles in tumor growth, remodeling of local stroma, bone marrow-derived cell education and recruitment, angiogenesis, invasion, suppression of the immune system and resistance to treatments. Exosomes released by both tumor cells and stromal cells play a role in the modulation of the primary tumor microenvironment, which affects cancer progression [30]. In the transfer process of exosome cargo, integrins and other receptors for target cell recognition are involved. Hoshino et al. [31] demonstrated that integrins are relevant in tissue organotropism of exosomes and that stromal cells in metastatic target organs uptake tumor exosomes in distinct ways. Several studies also suggest a role of exosomes in resistance to cancer treatment. In addition, upregulated biomarkers in exosomes detected in body fluids associated with resistance mechanisms could be potential predictive biomarkers used to monitor resistance. Tumoral exosomes can affect response to treatment in two ways: released drugs or the target of therapy inside them [32], and through specific cargo which is transferred to recipient cells. In colorectal cancer, patients receive chemotherapies that include platins, 5-Fluorouracil (5-FU) and targeted therapy. The acquisition of resistance to therapies can be due to inherent or acquired mechanisms. Thus, resistance to 5-FU in sensitive CRC cells is enhanced by exosomal p-STAT3 reducing caspase cascade activation [33] and may also be mediated by miR-196b-5p via targeting of SOCS1 and SOCS3 [34]. In targeted therapies, exosomes from cetuximab-resistant cells could induce resistance in sensitive cells by downregulation of Phosphatase and Tensin Homolog (PTEN), increase of phosphorylated AKT serine/threonine kinase 1 (AKT) levels and deregulation of UCA1 expression [35,36]. Exosomes released by CAFs, tumor-associated macrophages and other stromal cells from tumor microenvironment may also promote or inhibit tumor resistance to treatment [37].

### 2.1. Tumor Cell-Derived Exosomes’ Effect on Microenvironmental Cells

**Figure 1:**
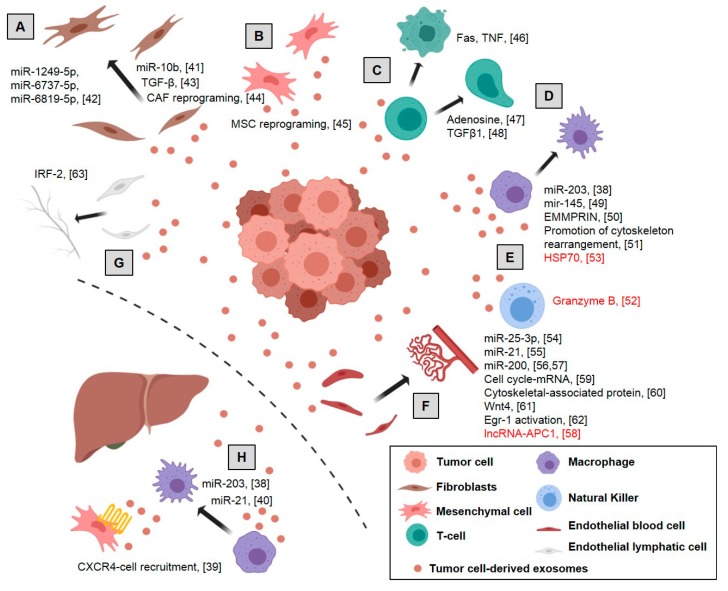
Role of tumor exosomes in colorectal cancer: Tumor exosomes act at different levels on the microenvironment, enhancing tumor progression and driving an inflammatory pre-metastatic niche, although in some cases an antitumor mechanism is also induced (red text): (**A**) induction of normal fibroblasts into Cancer-Associated Fibroblasts (CAFs), increasing the expression of myofibroblast markers and remodeling the extracellular matrix, (**B**) reprogramming mesenchymal stem cells to favor tumor growth and malignant progression, (**C**) induction of apoptosis of activated CD8+ T cells, negative regulation of T-cells and phenotypic alteration of the T cells to Treg, (**D**) polarization of M1 to M2 macrophages inducing a tumor-supporting phenotype in macrophages, (**E**) enhanced migration and reactivity of natural killer cells, (**F**) promotion proliferation and permeability of endothelial cells, increasing vascular permeability and angiogenesis, (**G**) modulation of lymphangiogenesis and (**H**) induction of the pre-metastatic niche by CXCR4-stromal cell recruitment, generating an immunosuppressive microenvironment. Dotted line represents separation between primary tumor and distal metastasis. References are shown in brackets [38,39,40,41,42,43,44,45,46,47,48,49,50,51,52,53,54,55,56,57,58,59,60,61,62,63]. Created with BioRender.com.

Exosomes from tumor cells play an important role in driving cancer progression by the modulation of surrounding cells and components. Therefore, tumor cell-derived exosomes induce neoangiogenesis, regulate phenotypes of fibroblasts and mesenchymal stem cells, remodel extracellular matrix components and jeopardize immune surveillance in the primary tumor and in the metastatic niche (Figure 1). 

#### 2.1.1. Pre-Metastatic Niche

The formation and preparation of the pre-metastatic niche in distant organs by tumor cells involve the orchestration of pro-metastatic signals of a variety of cytokines, growth factors and exosomes, which modulate the pre-metastatic sites before tumor invasion [64,65]. This preparation includes the modulation and recruitment of stromal cells in secondary organs which will form the metastases’ microenvironment cells and modulate the inflammation, immune response, angiogensis, organotropism and matrix remodeling for tumor cell invasion [66]. 

In CRC, the inclusion of miR-203 in tumor-derived exosomes facilitates the creation of the pre-metastatic niche in secondary organs by modulating immune surveillance [38]. In line with this, tumor-derived exosomes from CRC promote metastasis in distant organs by recruiting CXCR4-expressing stromal cells to develop a permissive metastatic microenvironment [39]. Moreover, an inflammatory microenvironment in the liver can also be induced by CRC exosomes containing miR-21. Exosomal miR-21 activates macrophages to a pro-inflammatory phenotype, forming an inflammatory pre-metastatic niche and, therefore, promoting liver metastasis [40].

#### 2.1.2. Fibroblasts Switch to Cancer-Associated Fibroblasts and ECM Remodeling

The miRNA cargo in exosomes has been widely studied. It plays an important role in the induction of Normal Fibroblasts into Cancer-Associated Fibroblasts. In colon cancer, tumor-derived exosomes containing miR-10b are transferred to fibroblasts in which Phosphatidylinositol-4,5-bisphosphate 3-kinase Catalytic Subunit Alpha (PIK3CA) expression is significantly suppressed by downregulation of PI3K/Akt/Mechanistic target of Rapamycin Kinase (mTOR) pathway activity. Thus, proliferation is reduced but the expression of myofibroblast markers is promoted, suggesting their reprogramming into CAFs [41]. In a similar way, exosomes derived from TP53-deficient colon cancer cells accelerate co-cultured fibroblast proliferation by the transfer of several microRNAs (miR-1249-5p, miR-6737-5p, and miR-6819-5p) that suppress TP53 expression in fibroblasts [42]. 

The induction of CAFs phenotype mediated by tumor exosome-mediated delivery of microRNAs has also been observed in many other tumors. Examples of this are the tumor transference of miR-9 and miR-105 in breast cancer [67,68], miR155 and miR-211 in melanoma [69,70], miRNA-21 in hepatocarcinoma [71] and ECM or miR-27a in gastric cancer [72]. In contrast, in ovarian cancer the epithelial cells transfer miR-124 to CAFs via exosomes, decreasing α-SMA and Fibroblast Activation Protein Alpha (FAP) expression and attenuated cell motility and thus reversing some traits of NFs [73].

During the last decade, many lncRNA larger than 200 bp have been identified as critical mechanisms of cancer biology regulation [74]. Although there are not data in CRC, in oral squamous cell carcinoma, tumor cells increased Lnc-CAF levels in stromal fibroblasts via exosomes to mediate stromal fibroblasts’ activation and raise the expression of CAF markers [75].

Proteins within tumor-derived exosome cargo are also involved in the reprogramming of normal fibroblasts to cancer-associated fibroblasts. In different tumor tissues, including colon cancer, cancer-derived exosomes differentiated into myofibroblasts, through Transforming Growth Factor beta (TGF-β) delivery [43]. In a recent study, Rai A et al. describe the role of primary and metastatic CRC tumor-derived exosomes in generating phenotypically and functionally distinct subsets of CAFs that may facilitate tumor progression. Thus, fibroblasts activated by primary tumor cancer exosomes were highly pro-proliferative and pro-angiogenic, and fibroblasts activated by metastatic cancer exosomes displayed a striking ability to invade the extracellular matrix through upregulation of pro-invasive regulators of membrane protrusion and matrix-remodeling proteins [44]. In addition, TGF-β delivered in cancer exosomes promote CAF differentiation in other different tumors like bladder cancer [76] or prostate cancer [77]. 

#### 2.1.3. Mesenchymal Stem Cell Reprogramming

Mesenchymal stem cells (MSCs), present in multiple tissues, are a significant component of the tumor microenvironment. They are adult multipotent cells, including chondrocytes, osteoblasts, adipocytes, and myocytes that are capable of self-renewal and differentiation in multiple lineages. Their involvement in cancer progression enhancing angiogenesis, epithelial-mesenchymal transition, metastasis, immunosuppression, and therapy resistance, has been extensively studied [78]. Different studies in many tumors described how malignant cells, through derived exosomes, modulate and reprogram MSCs, to induce microenvironmental changes that enhance tumor progression [79]. 

In colon cancer, tumor cells release high amounts of exosomes inducing morphological and functional changes in colonic MSCs. Thus, MSC after tumor exosome incubation showed atypical morphology, higher proliferation, migration, and invasion, ability to form spheroids, acidic extracellular environment, and a plasma membrane redistribution of vacuolar H+-ATPase with increased expression of Carcinoembryonic Antigen (CEA). Overall, these MSC changes caused by colon cancer cell-derived exosomes favor tumor growth and its malignant progression [45]. Similar mechanisms are also observed in renal [80] and lung tumors [81]. 

Although it is not described in CRC yet, different miRNAs and long ncRNAs packaged in tumor-derived exosomes, are involved in the reprogramming of MSCs, regulating their capacity for osteogenic and adipogenic differentiation in various tumors. For instance, lncRNA RUNX2-AS1 inhibits the osteogenic differentiation in multiple myeloma [82,83], and tumor-exosomal miRNAs promote the osteogenic differentiation of MSCs in breast and prostate tumors [84]. After internalization of lung cancer-derived exosomes by human adipose tissue-derived mesenchymal stem cells, TGF-β signaling or long-ncRNA and mRNA profiles are modulated in mesenchymal stem cell to inhibit adipogenesis [85,86]. 

In several tumors, such as breast, ovarian, gastric and prostate cancer, the enhancement of TGF-β_SMAD signaling in mesenchymal stem cells by tumor-derived exosomes has been observed. This signaling helps trigger a myofibroblastic phenotype, expressing αSMA by MSCs, which may enhance tumor proliferation and development [87,88,89,90]. 

#### 2.1.4. Immune Cells

Chronic inflammation and immune surveillance are closely related to cancer progression. The suppression or activation of immune cells by tumor cell-derived exosomes have been studied extensively in many tumor types [91]. 

In colon cancer, tumor exosomes bearing the Fas ligand and tumor necrosis factor-related apoptosis induce ligand-mediated apoptosis of activated CD8+ T cells as a mechanism of immune escape in cancer patients [46]. Moreover, in colorectal tumors, as well as in bladder, prostate and breast tumors, T-cells are also negatively regulated by the contribution of exosomal adenosine, which mediates the immune response in the tumor microenvironment [47]. In addition, TGF-β in CRC exosomes induce phenotypic alteration of the T cells to Treg-like cells by Smad signaling and inactivation of Mitogen-Activated Protein Kinase 9 (MAPK9) signaling. These Treg-like cells support notable tumor growth [48]. 

Tumor macrophages are normally classified as activated or M1 macrophages, or anti-inflammatory alternatively activated or M2 macrophages, depending on the expression of specific markers. Thus, the polarization status of M1 or M2 macrophages in the tumor burden determines their tumor-promoting or -supporting activity [92]. The *miR-203* in the CRC-derived exosomes is incorporated into monocytes promoting the reprogramming and differentiation of monocytes to M2-tumor-associated macrophages in metastatic CRC patients [38]. Similarly, CRC cells release miR-145 through exosomes being taken up by macrophage-like cells. Thus, macrophages, polarized into the M2-like phenotype through the downregulation of histone deacetylase 11, promote tumor progression [49]. High levels of the matrix metalloproteinase inducer, Basigin (Ok blood group) (EMMPRIN), were observed in exosomes isolated from cancer patients’ blood samples, including colorectal cancer patients. These exosomes induced a tumor-supporting phenotype in macrophages [50]. The proteome transported from CRC exosomes to macrophages was studied by means of a Stable Isotope Labeling with Amino Acids in Cell Culture (SILAC)-based mass spectrometry strategy. CRC exosomes transform cancer-favorable macrophages by rearrangement of the cytoskeleton [51]. 

The promotion of immune response and cytotoxic activity in colon cancer was also observed. The heat shock protein 70 on the plasma membranes of colon and pancreas cancer exosomes enhances the migration and reactivity of natural killer cells to stimulate and initiate apoptosis in tumors through granzyme B release [52]. In a similar way, exosomes derived from heat-stressed colon cancer cells contain heat shock protein 70, which strongly induces an antitumor immune response. These exosomes are potent stimulators of IL-6 secretion, which converts Tregs into Th17 cells with antitumoral effects [53]. However, it must be noted that the antitumoral role of Th17 is still controversial [93]. 

#### 2.1.5. Vascular Cells 

Tumor-derived exosomes are also involved in the regulation of the phenotype and functional reprogramming of endothelial and lymph cells. The expansion of new vessels is an early step in tumor development and necessary for tumor progression and metastases. The interaction of exosomes with endothelial cells to promote tumor angiogenesis has been described in several kinds of tumors [94].

Non-coding RNAs are also involved in the regulation of neoangiogenesis by tumor-derived exosomes in colon cancer. As in the case of microRNA, miR-25-3p is transferred from CRC cells to endothelial cells via exosomes promoting vascular permeability and angiogenesis through the regulation of VEGFR2, ZO-1, occludin and Claudin5 and the targeting of KLF2 and KLF4 [54]. Similarly, high levels of miR-21 in exosomes of several cancer cell types, including colon cancer, regulate proliferation, migration, and invasion of endothelial progenitor cells by IL6R targeting, and mediate vein thrombosis in patients with cancer [55]. Moreover, microRNA 200 contained in exosomes from colorectal cancer cells downregulates the expression of epithelial to mesenchymal transition-regulating transcription factors such as Zinc Finger E-box Binding Homeobox 2 (ZEB2), Snail Family Transcriptional Repressor 1 (SNAI), and Snail Family Transcriptional Repressor 2 SLUG in endothelial and lymphatic cells that modulate the resistance of endothelial barriers that resemble gates for tumor transmigration [56,57]. 

Inversely, colorectal cancer exosomes incorporate the long non-coding RNA-APC1, activated by APC regulator of WNT signaling pathway, to repress tumor angiogenesis. In fact, a decrease in this long non-coding RNA expression is positively associated with distant metastases and poor prognosis in colorectal cancer patients [58]. On the other hand, lncRNA H19 and HOX transcript antisense RNA (HOTAIR) are packaged into exosomes from tumor cells and transferred to endothelial cells to promote angiogenesis by expression of Vascular Endothelial Growth Factor (VEGF) in liver cancer and glioma cells [95,96]. Exosomal lncRNA regulator of Akt signaling Associated with HCC and RCC (lncARSR) released by resistant renal tumor cells mediates sunitinib resistance in tumor and endothelial cells, both targets of this kind of therapy, by competitively binding to miR-34 and miR-449 [97].

An mRNA analysis of colon cancer cell-derived exosomes demonstrated an enrichment of cell cycle-related mRNA, which promotes the proliferation of endothelial cells enhancing angiogenesis-related processes and thus tumor growth and metastasis [59]. The increase of endothelial permeability is also regulated by cytoskeletal-associated protein in colon cancer cell-derived exosomes. These proteins, mainly thrombin, are described as a key mediator of Ras Homolog Family Member A/Rho kinase (RhoA/ROCK) pathway activation, which induces amoeboid properties and destabilization of endothelial junctions [60]. In a similar way, the mesenchymal phenotype of endothelial cells is regulated by exosomal Wnt4 from colon tumor cells. The intake of exosomes with Wnt4 by endothelial cells increases β-catenin nuclear translocation and signaling, inducing proliferation and migration [61]. Egr-1 activation in endothelial cells is also observed after colon cancer-derived exosome treatment. This activation promotes migration of endothelial cells and thus angiogenic activity in colorectal cancer as well as in other pathological conditions, such as cardiovascular and neurodegenerative diseases [62]. 

In addition, cancer-derived exosomes can induce cancer-associated fibroblasts from endothelial and pericyte cells. The development of an in vitro 3D tumor, reproducing the tumor microenvironment in a microfluidic device model with human umbilical vein endothelial cells, showed endothelial-to-mesenchymal transition enhanced by melanoma-derived exosomes [98]. Moreover, the transition from pericytes to cancer-associated fibroblasts is also promoted by exosomes derived from gastric cancer cells by bone morphogenetic protein (BMP) transfer and PI3K/AKT and MAP kinse-ERK kinase/Extracellular Regulated MAP Kinase (MEK/ERK) pathway activation [99]. 

Furthermore, tumor cell dissemination is guided by the lymphatic network. IRF-2 in exosomes derived from colon cancer lines promotes the proliferation of lymphatic endothelial cells and the formation of the lymphatic network in the sentinel lymph node, increasing the frequency of F4/80+ macrophages and promoting Vascular Endothelial Growth Factor C (VEGFC) secretion [63]. Similarly, miR-221-3p, CXCR4 or podopladin have been related to lymphangiogenesis in other tumors [100,101,102]. 

### 2.2. Stromal Cell-Derived Exosomes’ Effects on Tumor and Stromal Cells 

**Figure 2:**
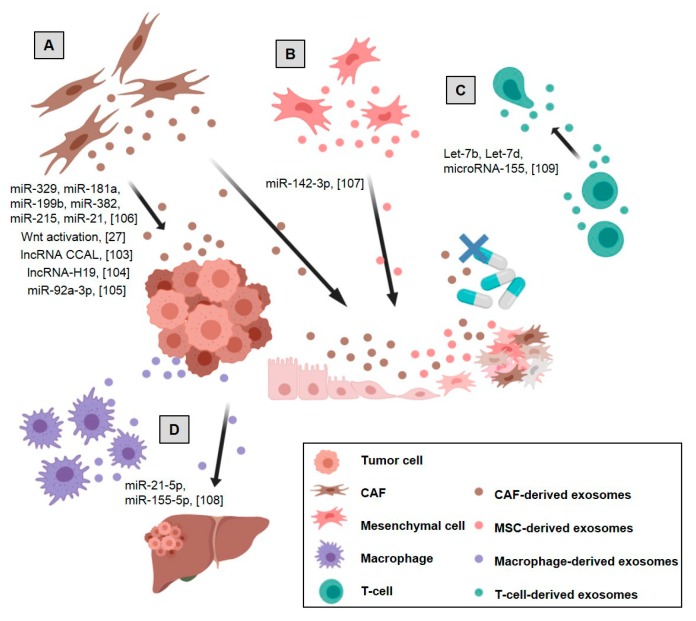
Stromal cell-derived exosomes’ effects on tumor and stromal cells in colorectal cancer: (**A**) CAF-derived exosomes promote tumor growth, migration, metastasis, and drug resistance, (**B**) mesenchymal stem cells (MSCs)-derived exosomes promote colon cancer stem cell-like traits, (**C**) Treg-derived exosomes inhibit Th1 immune response promoting immunosuppression, (**D**) M2 macrophage-derived exosomes promote tumor metastases. References are shown in brackets [27,103,104,105,106,107,108,109]. Created with BioRender.com.

Exosomes from stromal cells are also involved in driving cancer progression by the modulation of the tumor and surrounding cells. Thus, they are involved in tumor proliferation, stemness reprogramming, drug resistance, generation of metastatic niche and modulation of immunosurveillance (Figure 2).

#### 2.2.1. CAF-Derived Exosomes

Not long ago, the role of fibroblast-secreted exosomes in breast cancer progression [110] was reported for the first time. Since then, different studies have investigated the pleiotropic effect of CAF as an active source of exosomes. In vitro and in vivo models have shown that exosomes secreted by CAF promote tumor growth, migration, metastasis, and drug resistance through various mechanisms [111]. 

It has been suggested that the acquisition of cancer stemness could cause chemoresistance in CRC [112]. Recent functional studies have demonstrated that CAFs enhance the cell stemness properties of colorectal cancer cells. This increases resistance to oxaliplatin and 5-FU treatment and may be mediated by CAF exosomes activating the Wnt signaling pathway in recipient cells [27]. 

Various non-coding RNAs in the exosome cargo of colorectal CAFs have been described as participants in chemoresistance. In this way, colorectal cancer-associated lncRNA is transferred from CAFs to CRC cells via exosomes that activate the β-catenin pathway by directly binding to mRNA stabilizing protein HuR, leading to an increase of β-catenin and eventually conferring oxaliplatin chemoresistance [103]. Likewise, CAF-derived exosomes’ delivery of lncRNA-H19 to colon cancer cells induces stem cell properties and drug resistance by Wnt family (Wnt)/β-catenin signaling activation [104]. In the case of microRNAs, the transfer of exosomal miR-92a-3p to tumor cells activates the Wnt/β-catenin pathway and prevents mitochondrial apoptosis by FBXW7 and MOAP1 inhibition, contributing to cell stemness, epithelial-mesenchymal transition, metastasis and 5-FU/Oxaliplatin resistance. In this study, high levels of miR-92a-3p in exosomes of plasma from CRC patients were associated with metastatic disease and chemotherapy resistance [105]. Moreover, an exosomal CAF signature consisting of microRNAs 329, 181a, 199b, 382, 215 and 21 were shown to be involved in the modulation of tumor cell microRNA levels which will have an impact on proliferation and chemoresistance in CRC [106]. 

Exosomes released by fibroblasts cooperate to induce resistance of cancer cells against cytotoxic drugs in other tumor types as well. This was seen in the pancreas, [113], head and neck [114], breast [115] and ovarian cancer [116]. In addition, CAF-derived exosomes regulate survival and proliferation of epithelial cells and boost cancer progression in head and neck [117] and pancreas cancer [118]. Migration and invasion are also stimulated by CAF-derived exosomes in oral squamous cell carcinoma [119], osteosarcoma [120] and endometrial metastasis [121]. Eventually, CAF-derived exosomes also regulate stemness capabilities and the epithelial-mesenchymal transition phenotype in oral squamous cell carcinoma [122], breast [123,124] and lung cancer [125].

Although it is not described in the CRC context, it should be pointed out that some other molecules cargo are downregulated in CAF-derived exosomes. This could affect the metastatic phenotype of cancer cells as it was seen in oral squamous cell carcinoma (miR-34a-5p) [126] and in hepatocellular carcinoma (miR-320a) [127].

Additionally, it has been demonstrated that CAF-derived exosomes contain metabolites in their cargo that are used by cancer cells in their central carbon metabolism to promote cancer growth under nutrient deprivation or nutrient-stressed conditions. Thus, CAF-derived exosomes supply amino acids, lipids, and tricarboxylic acid cycle intermediates, inhibiting mitochondrial oxidative phosphorylation and thereby increasing glycolysis and glutamine-dependent reductive carboxylation in pancreatic cancer cells [128,129].

#### 2.2.2. Mesenchymal Stem Cell-Derived Exosomes

In the tumor microenvironment, Mesenchymal Stem Cells (MSCs) have a dual effect on cancer progression: they stimulate or inhibit growth in different cancer types [130]. MSCs are highly efficient producers of exosomes carrying individual messages to fibroblasts, endothelial cells, immune cells and tumor cells [79]. Like MSCs, MSC-derived exosomes can have either anti- or pro-tumorigenic effects [131]. 

In colon cancer, exosomes from bone marrow mesenchymal stem cells promote colon cancer stem cell-like traits via miR-142-3p by Numb targeting and promotion of the Notch signaling pathway [107]. Cell growth, stemness, metastasis, and chemoresistance are also activated by MSC-derived exosomes in gastric cancer [132,133,134,135], multiple myeloma [136], nasopharyngeal tumor cells [137], oral squamous carcinomas [138] and breast cancer [139,140,141].

In contrast, several studies show the suppressive role of MSC-derived exosomes in tumor progression. For instance, in hepatocellular carcinoma, exosomes derived from MSCs inhibit tumor growth, progression, and metastasis by enhancing tumor apoptosis and downregulating angiogenesis, epithelial-mesenchymal transition and tumor invasiveness [142]. Similarly antitumoral roles of MSC-derived exosomes are observed in ovarian [143], breast [144,145], leukemia [146,147] and melanoma cells [148].

#### 2.2.3. Endothelial Cell-Derived Exosomes

Enhancement and maintenance of angiogenesis are needed for cancer progression. Some studies revealed the role of exosomes in communications between endothelial cells, endothelial progenitor cells, and stromal cells and their involvement in tumor angiogenesis regulation. For instance, the miR-214 encapsulated in endothelial-derived exosomes plays an important role in the stimulation of neighboring endothelial target cells [149]. Although there is no information in CRC, endothelial-derived exosomes were shown to modulate tumor proliferation, migration and chemoresistance in breast cancer [150,151], nasopharyngeal carcinoma cells [152], hepatocellular carcinoma [153] and small-cell lung cancer [154].

#### 2.2.4. Immune Cell-Derived Exosomes 

New evidence displays the impact of exosomes derived from immune cells, like macrophages, natural killer cells and the different types of T cells, on the phenotype and function of tumor cells. A better understanding of the cross-talk between immune cell-derived exosomes and the tumor microenvironment could provide new insights into novel anti-cancer strategies [155].

In colorectal cancer, M2 macrophages release exosomes packing miR-21-5p and miR-155-5p to target the BRG1 sequence in colorectal cancer recipient cells, which promotes tumor metastases [108]. The role of macrophage-derived exosomes in tumor progression has been studied in other tumor types too. As an example, in gastric cancer, it was seen to be involved in chemoresistance [156] and cytoskeleton remodeling, although some other antitumoral effects were also observed [157]. In breast, pancreatic and ovarian tumors, exosomes from macrophages promote proliferation, metastasis, angiogenesis, immunosuppression, and impairment of drug sensitivity [158,159,160,161,162,163].

Exosomes derived from natural killer cells express the typical protein markers of natural killer cells, such as the Fas ligand. These exosomes exert cytotoxic effects against tumor cells and activate immune cells, which shows their potential use as part of an immunotherapeutic strategy [164,165].

T cells also release exosomes involved in cell-mediated communication and in the regulation of the immune response and thus in tumor progression [166]. The transfer of Let-7b, Let-7d, and microRNA-155 into Tregs-derived exosomes regulates the inhibition of the Th1 immune response, thus promoting immunosuppression [109]. Exosomes derived from T cells promote melanoma cell invasion [167], esophageal carcinoma metastasis [168] and impairment of the anti-tumor potential of non-exhausted CD8+ T cells in hepatocellular carcinoma [169]. 

## 3. Microenvironment-Derived Components as Liquid Biopsy Biomarkers

The tumor microenvironment releases many promising circulating soluble factors that could be used as biomarker tools for oncology clinical practice (Table 1). However, sometimes it is difficult to assess the specific origin of these factors since they could be released by different cells, including tumor cells. 

### 3.1. Soluble Factors

A new concept is gaining attention in recent years. Extracellular matrix remodeling is a necessary step in tumor development [26]. During this remodeling, many ECM components are released into peripheral blood that represents potential biomarkers for the diagnosis, prognosis and treatment response of different types of cancer patients [170].

Matrix metalloproteinase is involved in tumor progression and matrix remodeling [26]. In colon cancer, many studies confirm its diagnostic value [170]. Similarly, collagen fragments from the formation and degradation of ECM by fibroblasts could be released to circulation and evaluated as diagnostic and prognostic biomarkers in many tumors [188]. In colon cancer, various collagens show greater concentration in patients than in healthy controls [171,172]. However, their ability to discriminate between malignant and non-malignant lesions is not well established in all studies [173]. Similarly, endostatin levels differ in advanced-stage cancer patients, but their diagnostic value in healthy patients is not clear [174]. In addition, the combination of TIMP-1 and different metalloproteinases predict patient survival in colon cancer patients [175]. 

### 3.2. Exosomes as Biomarkers

Various efforts have attempted to provide a detailed description of CAF exosome composition that would be useful for future mechanistic and biomarker studies (Table 1). In this context, RNA sequencing analysis of non-coding RNAs in exosomes from normal fibroblasts and CAFs in colorectal cancer reveals ncRNA regulatory elements specifically packaged in CAF-derived exosomes that might play a role between CAFs and cancer cells and/or other stromal cells [18]. Also in CRC patients, high expression of CAF-exosomal miR-92a-3p is detected in serum samples linked with metastasis and chemotherapy resistance [105]. Therefore, a CAF-exosomal microRNA signature modulating tumor cell proliferation and resistance is proposed as a good potential biomarker [106]. In addition to this, cargo from CAF-derived exosomes is also associated with tumor development and patient survival in oral squamous cell carcinoma [189] and in head and neck cancer [114]. 

The exosome cargo of other microenvironment cells also shows their potential as cancer biomarkers. For example, circulating exosomal miRNAs derived from macrophages or endothelial cells, are related to recurrence and chemotherapy treatment in ovarian [163] and breast cancer patients [150].

Despite the large number of studies evaluating exosomal RNA, results have not yet been transferred to the clinic. This is due to several key points: lack of uniformity in the studies and technical difficulties using this type of sample. There is no standardization about methods of isolation and purification of exosomes and other extracellular vesicles, affecting the interpretation of results. Extracellular vesicles isolated by ultracentrifugation protocols or commercial kits may be contaminated with extracellular RNA derived from non-extracellular vesicles, such as those in protein (AGO2), immune and lipoprotein (HDL) complexes [190,191,192]. International Society for Extracellular Vesicles (ISEV) has proposed required criteria and the minimal characterization of extracellular vesicles to perform adequate studies. [193,194]. Regarding isolation methods, each type shows the pros and cons, affecting RNA yield and identity. Even type of sample, collection tube, storage conditions and processing of exosomes can influence the results. For example, serum contains higher numbers of EV released by platelets than plasma. An extensive review comments these points, indicating best practices to obtained reproducible results [195]. Moreover, other considerations must be taken into account, such as recently two subpopulations of exosomes have been described, showing different protein, RNA cargo and effects on recipient cells [196]. 

### 3.3. Tumor-Related Circulating Cells 

Besides circulating tumor cell lines (CTCs), different tumor-related circulating cells, like cancer-associated macrophage-like cells, circulating endothelial cells or cancer-associated fibroblast/mesenchymal cells have clinical value in cancer diagnosis, prognosis, and treatment response. The analysis of CTCs and these circulating cells could be used to improve the sensitivity and specificity of tumor biomarkers in liquid biopsy (Table 1). 

#### 3.3.1. Circulating Endothelial Cells

Circulating endothelial cells (CECs), like endothelial progenitor cells, are rarely observed in healthy controls but are often detected in cancer patients. Their use in treatment response monitoring is a promising clinical tool [197].

Many cancer treatments include anti-angiogenic therapies to block tumor neoangiogenesis, which is a pivotal mechanism in cancer development. Although the identification and quantification of these cells are not well standardized, they represent a promising tool for monitoring the clinical response and outcome of patients, including colon cancer patients [176,177]. However, the outcome prediction value of CECs or endothelial progenitor cells in colorectal cancer patients with or without anti-angiogenic therapy remains unclear [178].

To characterize the difference between CECs from cancer patients and healthy controls, CD276 is described as a good candidate in advanced colorectal and other cancer patients [179]. Similarly, the transcriptomic analysis showed differences between healthy volunteers from treatment-naïve as well as pathological early-stage colorectal cancer patients [180]. Therefore, the potential of using CECs as screening markers for colorectal cancer diagnosis is really encouraging.

The putative role of CECs as cancer biomarkers has been recently reported in prostate cancer. Thus, the accuracy of CECs as a screening biomarker has much better predictive value than classic PSA tests [198]. Similarly, in lung cancer patients, CECs and microparticles are taken as diagnostic and prognostic biomarkers [199,200].

As previously commented, many studies assess the prognostic value of CECs. Supporting the preclinical data, many clinical studies also show the utility of CECs as cancer biomarkers. In colorectal cancer patients, the quantification of CECs could improve the identification of early predictors of response to bevacizumab combined with FOLFOX (FOL–Folinic acid (leucovorin), F–Fluorouracil (5-FU), OX–Oxaliplatin (Eloxatin)/OXXEL (Oxaliplatin plus Xeloda) [181]. Similarly, other studies have assessed the predictive value of CECs for anticancer therapies in urothelial [201], breast [202], lung [203] and renal carcinoma [204].

#### 3.3.2. CAFs and Mesenchymal Circulating Cells 

Although there is no data in colorectal cancer, surprisingly, CAFs have also been observed in the blood circulation of various types of cancer patients. In breast cancer, circulating CAFs are detected in the peripheral blood of patients, predominantly in those with metastatic disease [205]. In a similar way, in prostate cancer, circulating fibroblast-like cells are detected in almost 60% of metastatic patients, but not in patients with a localized tumor or in healthy donors, thus providing diagnostic information [206]. In addition, serial blood analysis in breast and lung cancer patients, when compared with healthy donors, identifies circulating tumor cells, circulating mesenchymal cells, putative circulating stem cells and circulating endothelial cells, which provides evidence for its use as a cancer biomarker [207]. 

#### 3.3.3. Circulating Immune Cells

Though very rare, some studies describe the presence of macrophage-like cells in the blood circulation of cancer patients. In breast cancer, circulating cancer-associated macrophage-like cells are observed in more than 90% of known cancer patients but none in healthy volunteers, [208] and is associated with the breast cancer stage [209]. Along the same lines, the following of RAD50 and PD-L1 expression in cancer-associated macrophage-like cells is suggested as a surrogate for tracking adaptive changes in immunotherapeutic targets in lung cancer patients undergoing radiotherapy [210]. Recently, a new cell type has been described in circulation, tumor-derived circulating hybrid cells [211] which derive from tumor-macrophage fusion and retain properties of both cell types. These cells play a role in disease progression, and a high number of them are associated with advanced stages and poor overall survival. 

Most studies of T-cells in cancer immunity have been performed within tumors. However, these cells can also be analyzed in circulating peripheral blood and could be used as potential biomarkers in liquid biopsy. A significant correlation has been observed between circulating CD3^+^ and CD4^+^ T-cells and CD3^+^ and CD8^+^ Tumor-infiltrating lymphocytes from CRC tumors [182]. In CRC patients, the proportion of Treg cells in the Th cells subgroup is greater in peripheral blood mononuclear cells than in healthy controls [183]. Moreover, through analysis of tumor-infiltrating lymphocytes signatures based on cytokine-induced phosphorylated Signal Transducer and Activator of Transcription (STAT) proteins (CIPS signature), in peripheral blood mononuclear cells showed a signature partially represented in tumor-infiltrating lymphocytes. Thus, cytokines such as IL-6 and IL-10 modify phosphorylation in STAT proteins both in tumor-infiltrating lymphocytes and in peripheral blood mononuclear cells. IL-6- and IL-10-induced p-STAT3 signatures in peripheral blood mononuclear cells may be used as diagnostic biomarkers to distinguish CRC patients from healthy individuals with a sensitivity and specificity of 91% and 88%, respectively. Similarly, another study in colorectal cancer patients showed higher percentages of Tregs, myeloid-derived suppressor cells and neutrophil-to-lymphocyte ratio in the blood of patients than in healthy controls [184]. In addition, several immune checkpoint molecules were deregulated in the peripheral blood immune compartment, for example, PD-1 in T cells. Other proteins over-expressed in circulating T cells, such as Laryngeal Adductor Paralysis (LAP) and FOXP3 in Tregs and TIM3 and PD-1 in CD8^+^ T cells, are associated with CRC diagnosis and with metastases [185,186]. Mucosal-associated invariant T cells secrete cytokines, thus increasing anti-tumor responses through natural killer cells and CD8^+^ T cells. These cells were fewer in blood from colorectal cancer patients than in healthy donors and correlated inversely with N stage [187]. These relations were not found in other tumor types, which do not show a mucosal barrier. Moreover, circulating mucosal-associated invariant T cells express high levels of CCR6 and CXCR6 in patients. Therefore, these cells could reflect the degree of cancer progression in CRC patients.

## 4. Therapeutic Applications 

Elimination of exosomes could help to inhibit tumor progression, metastasis, and resistance to treatment. The Aethlon ADAPT^TM^ System (adaptive dialysis-like affinity platform technology) is a therapeutic hemofiltration process, which depletes exosomes from the blood circulatory system using affinity plasmapheresis [212]. This strategy does not introduce toxicity or interaction risks. Another approach using exosomes to inhibit metastasis is M-Trap technology [213]. M-Trap is a 3D scaffold, which forms an artificial premetastatic niche that mimics the extracellular matrix. Exosomes obtained from effluxions are embedded into the inserted scaffold and are used as a substrate to capture disseminated tumor cells. The surgical removal of M-Trap disrupted the process of metastasis.

In addition, exosomes may be used as drug delivery vehicles through the manipulation of their cargo. Exosomes can be loaded with specific protein, miRNA or short interference RNA in several ways: (a) transfection of the relevant gene into the donor cells, although loading efficiency and specificity are not stable, (b) fusion of target with a constitutive protein of exosomes, (c) use of specific modifications in the target involved in loading, such as ubiquitination, and (d) mechanical methods, such as sonication, saponin permeabilization, and electroporation [214]. MSCs are widely used in the therapeutic production of exosomes due to their high and reproducible extravesicules production [215]. For example, exosomal Tumor Necrosis Factor (TNF)-related apoptosis-inducing ligand produced by MSC induces apoptosis in cancer cell lines, even in “TNF-related apoptosis-inducing ligand”-resistant cancer cells [216]. Packaging of miR-122 into MSC-derived exosomes also enhances the imatinib and sorafenib chemosensitivity of human leukemia and hepatocarcinoma cells [146,147]. Yeast cytosine deaminase::uracil phosphoribosyl transferase suicide fusion gene (yCD::UPRT) loaded into MSC exosomes acts as a suicide gene. When exosomes were internalized in tumor cells in the presence of the prodrug 5-fluorocytosine, intracellular conversion of the prodrug to 5-FU induced tumor cell death [217]. Exosomes derived from M1-polarized macrophages show therapeutic effects. M1 exosomes may be used as immunopotentiators for a cancer vaccine, as they were more potent than CpG oligonucleotide in a melanoma study [218]. In another study, M1 macrophage exosomes repolarized M2 tumor-associated macrophages to M1, inducing anti-tumor immune responses [219]. Specific short hairpin-RNAs to KRASG12D were loaded into exosomes delivered by normal fibroblast-like mesenchymal cells. Treatment with these exosomes decreased tumor growth and increased overall survival in a mouse model of pancreatic cancer [220]. 

As a delivery system, exosomes can be loaded with drugs, improving their extravasation in tumors. Doxorubicin and Paclitaxel can be loaded into exosomes, decreasing tumor progression in CRC and other tumor types [221]. Synthetic exosome mimetics loaded with paclitaxel also inhibited breast tumor growth and were found to be therapeutically efficient in both in vitro and in vivo experiments [222]. Paclitaxel loaded into M1 macrophage-derived exosomes showed antitumoral efficacy in lung cancer cells [223,224].

## 5. Concluding Remarks

Colorectal cancer is one of the most common malignancies and one of the primary causes of tumor-related deaths worldwide. In the USA alone, around 150,000 new cases of colorectal cancer will be diagnosed in 2019 and 50,000 patients will die as a consequence of tumor progression. Although the 5-year survival rate is around 90% in localized tumors, only 10–15% will survive at 5 years with metastatic disease [225]. This is why the early diagnosis of the disease is one of the main focuses of studies. In addition, prognostic and treatment response biomarkers have also been extensively studied in recent decades. 

The liquid biopsy is successful as a new non-invasive method for early detection and tracking of biomarkers, especially in blood. The identification of liquid biopsy-based biomarkers that discriminate between asymptomatic cancer patients and healthy controls, as well as between tumors with different prognoses, and that monitor treatment response, is of unquestionable clinical utility. 

In addition, the tumor microenvironment and biological cross-talk between tumor and stromal cells in the tumor microenvironment and at distant locations are rising in importance as potential mechanisms of the tumor progression. Thus, the different signals derived from the tumor microenvironment, such as soluble factors, circulating cells and most exosomes, which can be detected in blood samples, are emerging as the new biomarkers for cancer diagnosis, prognosis, treatment response prediction, and treatment monitoring. Moreover, the description of exosome cargo and function led to the development of new therapeutic tools as vehicle systems in which exosome cargo is ectopically manipulated to deliver protein, miRNA or short interference RNA, so as to specifically regulate target genes involved in tumor progression or anti-tumor drugs to improve cancer treatments.

## Figures and Tables

**Table 1:** Summary of the Microenvironment-derived components with value as liquid biopsy biomarkers in colorectal cancer (CRC) patients.

Soluble Factors in Blood	Involvement	References
Matrix metalloproteinase	With diagnostic value	[26,170]
Collagens	Higher levels in CRC patients than in healthy controls	[171,172,173]
Endostatin	Higher levels in advanced CRC patients	[174]
TIMP-1 + metalloproteinases	Prediction of patients survival	[175]
**CAF Exosomes as Biomarkers**		
Non-coding RNAs signature	Regulatory elements specifically packaged in CAF-derived exosomes	[18]
miR-92a-3p	Higher levels associated with metastases and chemoresistance	[105]
microRNA signature	Regulation of tumor cell, proliferation, and chemoresistance	[106]
**Circulating Endothelial Cells (CECs)**		
Identification and quantification of CECs	Monitoring clinical response and outcome	[176,177,178]
CD276	Increase expression in tumor-derived endothelial cells	[179]
Transcriptomic analysis	Differentiation between healthy controls and CRC early stages	[180]
Quantification of CECs	Identification of early predictors of response to bevacizumab and FOLFOX/OXXEL	[181]
**Circulating Immune Cells**		
Treg, myeloid-derived suppressor cells, and neutrophil-to-lymphocyte rate	Identification of CRC patients versus healthy controls	[182,183,184]
Immune checkpoints and clinical outcome	Association with diagnosis and metastasis	[185,186]
Mucosal-associated invariant T cells	Increase number in CRC patients versus healthy controls	[187]

## Abbreviations

5-FU	5-Fluorouracil
CAFs	Cancer-Associated Fibroblasts
CECs	Circulating Endothelial Cells
CRC	Colorectal Cancer
CTCs	Circulating Tumor Cells
ctDNA	circulating tumor DNA
ECM	Extracellular Matrix
lncRNAs	long non-coding RNAs
miRNAs	micro RNAs
MSCs	Mesenchymal Stem Cells
TGF-β	Transforming Growth Factor beta
